# Age-differentiated impacts of digital health literacy and anxiety syndrome on hypochondriasis during the COVID-19 pandemic in South Korea: a cross-sectional study

**DOI:** 10.7586/jkbns.25.017

**Published:** 2025-08-14

**Authors:** Myung-Ock Chae, Hae Ok Jeon, Ahrin Kim

**Affiliations:** Department of Nursing, Cheongju University, Cheongju, Korea

**Keywords:** Age groups, Anxiety, COVID-19, Health literacy, Hypochondriasis

## Abstract

**Purpose:**

This study aimed to determine the impact of coronavirus disease 2019 (COVID-19) related digital health literacy and COVID-19 anxiety syndrome on hypochondriasis according to age groups among Korean adults.

**Methods:**

The study included 242 participants, including 97 young adults, 72 middle-aged adults, and 73 older adults, with an even distribution of age groups. The data were analyzed using SPSS version 27.0, employing descriptive statistics, the Fisher-Freeman-Halton exact test, one-way analysis of variance, the Scheffe test, and multiple linear regression analysis.

**Results:**

COVID-19 anxiety syndrome (β = 0.28, *p* = .009) was found to be a significant factor influencing hypochondriasis among young adults, but this model was found to have no statistically significant explanatory power; COVID-19 anxiety syndrome (β = 0.47, *p* < .001), and searching as a component of digital health literacy (β = −0.42, *p* = .018) were significant factors among middle-aged adults (29% explanatory power; F = 3.12, *p* = .001); and COVID-19 anxiety syndrome (β = 0.40, *p* < .001) and diagnosis of chronic disease (β = 0.32, *p* = .006) were significant factors among older adults (24% explanatory power; F = 2.58, *p* = .006).

**Conclusion:**

Measures to reduce COVID-19 anxiety syndrome in adults of all ages are needed to manage COVID-19-provoked hypochondriasis. In particular, differentiated strategies to improve COVID-19-provoked hypochondriasis according to age groups need to be developed, taking into account searching as a component of digital health literacy among middle-aged adults and diagnosis of a chronic disease among older adults.

## INTRODUCTION

Coronavirus disease 2019 (COVID-19) is a novel infectious disease that first emerged in December 2019 causing millions of deaths worldwide, with 34,572,554 cumulative cases and 35,605 cumulative deaths in South Korea [1]. COVID-19 was most prevalent in young adults in their 20s and 30s with 10,078,869 cases, while the elderly aged 60 and above had the lowest prevalence with 7,207,868 cases [1]. The proportion of COVID-19 deaths by age groups was lowest among those aged 40 and younger, at 2.2%, followed by 11.3% among those aged 60 and above, 22.7% among those aged 70 and above, and 59.7% among those aged 80 and above, with mortality rates rising sharply with age [2].

The COVID-19 pandemic had a profound impact on the way people live, including social distancing and home isolation [3]. As a result, most people have experienced uncertainty about their future, which negatively impacted the physical and mental health of people of all ages [3]. In particular, young adults perceived the situation caused by COVID-19 as a major threat to their personal economic situation, while older adults perceived it as a major threat to their health, indicating a clear difference in perceptions of COVID-19 by age groups [4]. In fact, older adults had a higher fatality rate from COVID-19 compared to younger adults [2], and adherence to precautions to prevent the spread of COVID-19 was lowest in the 18-29 age group and highest in the 60 and older age group [5].

Health literacy refers to an individual's ability to critically evaluate available health information [6]. With the majority of COVID-19 related information available online as social distancing progressed [7], digital health literacy emerged as an important concept to assess the quality and appropriateness of internet information for obtaining and applying information to make health-related decisions [7]. COVID-19 related digital health literacy influenced their health information utilization and preventive behaviors [8], and the process of digital health literacy utilization provided psychosocial support for through online communities [9]. However, there are inequalities in digital health literacy across socioeconomic levels [10], and there are differences in the level of digital health literacy and related behaviors by age groups.

The COVID-19 pandemic has shown that people experience anxiety for a variety of reasons, including contact with people with COVID-19 [11], COVID-19 lockdowns [12], COVID-19 vaccination [13], physical and cognitive decline, loss of family and friends [14], COVID-19 testing, and death [15], with differences across age groups [11-13]. Anxiety has been linked to coronaphobia [16], and higher levels of anxiety have been associated with greater feelings of helplessness in coping with stress [17]. Anxiety can manifest as a complex of symptoms, and the cluster of maladaptive coping responses such as avoidance, checking, and worry in response to fear or threat of COVID-19 has been termed COVID-19 anxiety syndrome [18,19]. Therefore, a more specific and clear assessment of anxiety as a broader symptom, utilizing a tool of COVID-19 Anxiety Syndrome, a concept that includes COVID-19 anxiety and its associated negative coping responses, may be better suited to analyze differences by age groups.

The tendency to misread physical signs, the conviction that one already has a serious illness, and the refusal to regular medical reassurance are all characteristics of hypochondriasis [20]. Health anxiety, a subset of hypochondriasis, has been shown to increase the threat of COVID-19 related death [21], and watching COVID-19 related news, browsing social media, and acquiring inaccurate information has been shown to exacerbate health anxiety [22,23]. Prior research has shown that higher preference for gathering information is associated with more COVID-19 worry [3], and older age is associated with more COVID-19 worry [3]. In fact, the phenomenon of repeatedly searching the internet for health information due to excessive worry about health is called cyberchondria [24], which has also been shown to vary by age [25]. This suggests that hypochondriasis is closely related to COVID-19 anxiety and digital health literacy, and that age differences need to be considered.

However, to date, it was difficult to find a comprehensive study that compared differences in COVID-19 digital health literacy, COVID-19 anxiety syndrome, and hypochondriasis by age groups, and identified factors that influence hypochondriasis by age groups, including COVID-19 digital health literacy and COVID-19 anxiety syndrome. Therefore, this study aimed to provide a basis for developing nursing interventions by comparing the levels of COVID-19 digital health literacy, COVID-19 anxiety syndrome, and hypochondriasis by age groups among Korean adults, and identify factors affecting hypochondriasis by age groups. It can also help build a customized, age-specific strategy for managing infectious disease-provoked hypochondriasis in the event of another pandemic in the future.

## METHODS

### 1. Study design

This study is a descriptive survey study to determine the impact of COVID-19 related digital health literacy and COVID-19 anxiety syndrome on hypochondriasis according to age groups among Korean adults.

### 2. Participants

The participants were Korean adults above the age of 19, comprising young adults (19~44 years old), middle-aged adults (45~64 years old), and older adults (≥ 65 years old). To measure the research variable of digital health literacy, participants were limited to those who were able to use the internet to search for information through verbal and written explanations. The survey was conducted with an equal number of participants in each age group. To avoid skewing the sample toward occupations and education levels, we recruited participants from diverse organizations, including large and small businesses, schools, senior centers, and churches. The minimum sample size to conduct this study was analyzed using the G*power 3.1.9.2 program [26]. Based on linear multiple regression, 14 predictor variables were included (sex; education level: ≤ junior high school; High school, marital status; employment; living arrangement; diagnosis of chronic disease; COVID-19 testing experience; COVID-19 self-isolation experience; COVID-19 related health literacy-searching, expressing, evaluating, using, and COVID-19 anxiety syndrome), with a significance level of .05 and a medium effect size (0.15); the minimum sample size to maintain a power of .80 was 55 per age group. The effect size was set based on a medium effect size, which is a commonly used approach for estimating sample size in social science and psychological research. This decision was informed by the effect size reported in a previous regression analysis study on stress coping among older adults during the COVID-19 pandemic [17]. Considering the COVID-19 situation and the preferred data collection method (online vs. offline survey) for each age group, we set an expected dropout rate of 30% and proceeded with data collection. In conducting a comparative study by age group, prior research has indicated that the ability to search for information online tends to decline with increasing age [9,27]. Taking this into account, along with the potential for dropout and incomplete responses, a dropout rate of 30% was established for this study. Of the 244 completed questionnaires, we excluded two questionnaires(offline survey) with insufficient responses and used the final 242 (97 young adults, 72 middle-aged adults, and 73 older adults) for the final analysis.

### 3. Instruments

#### 1) Socio-demographic characteristics, health, and COVID-19 related characteristics

Sociodemographic characteristics included questions about age, sex, education level, marital status, employment, living arrangement, and economic status. Health-related characteristics included questions about the diagnosis of chronic disease, subjective health status, COVID-19 testing experience, COVID-19 confirmed experience, after-effects of COVID-19, COVID-19 self-isolation experience, and experience with a family member who died of COVID-19.

#### 2) COVID-19 related digital health literacy

To measure COVID-19 related digital health literacy, the Korean version of the Digital Health Literacy Instrument (DHLI) by Chun et al. [7], which is based on the DHLI by van der Vaart and Drossaert [27], was used. The tool consists of 12 questions, with three questions each for the following four sub-domains: “searching,” “expressing,” “evaluating,” and “using” information, with responses on a four-point scale ranging from “very difficult” to “very easy.” Higher scores indicate a higher level of COVID-19 related digital health literacy in each domain. In the study by Chun et al. [7], Cronbach’s α was reported as .91, with subdomain values of .84 for searching, .86 for expressing, .77 for evaluating, and .84 for using. In the present study, Cronbach’s α was .94, with subdomain values of .91 for searching, .89 for expressing, .82 for evaluating, and .86 for using.

#### 3) COVID-19 anxiety syndrome

To measure COVID-19 anxiety syndrome, the COVID-19 Anxiety Syndrome Scale (C-19ASS), developed by Nikčević and Spada [19], was translated into Korean and then back-translated. To ensure expert validity of the translation while reflecting the cultural context of Korea, the translated items were reviewed by one bilingual nursing professor. Furthermore, content validity was assessed by three nursing professors with extensive research experience in anxiety and psychological health, focusing on the appropriateness, clarity, and cultural relevance of each item. The tool consists of nine questions, six for Perseveration (C-19ASS-P) and three for Avoidance (C-19ASS-A), measured on a 5-point Likert scale. Higher total scores indicate higher levels of COVID-19 anxiety syndrome. In Nikčević and Spada’s study [19], Cronbach's α for C-19ASS-P was 0.86 and for C-19ASS-A was 0.77. The Cronbach's α for the Korean version of the instrument developed in this study was: C-19ASS-P .82 and C-19ASS-A .77.

#### 4) Hypochondriasis

To measure hypochondriasis, we used the Illness Attitudes Scale developed by Kellner [28] and translated into Korean by Yi [29]. The tool is organized into eight subscales with a total of 27 questions on a 5-point Likert scale ranging from 0 to 4. The eight subtopics are: Worry about illness (WI), Health habits (HH), Hypochondriacal beliefs (HB), Thanatophobia (TH), Disease phobia (DP), Bodily preoccupations (BP), Treatment experiences (TE), and Effects of symptoms (ES). Higher scores indicate a greater severity of hypochondriasis in each domain. In Yi’s study [29], Cronbach’s α was .86, with subscale values of .75 for WI, .62 for HH, .80 for HB, .73 for TH, .82 for DP, .62 for BP, .74 for TE, and .84 for ES. In the present study, Cronbach’s α was .92, with subscale values of .88 for WI, .74 for HH, .77 for HB, .79 for TH, .87 for DP, .71 for BP, .81 for TE, and .89 for ES.

### 4. Data collection

Data collection was conducted from August 16 to September 16, 2022, using a combination of online and offline surveys. This mixed-method approach was adopted in consideration of the COVID-19 situation and the preferred data collection methods across different age groups. Participants were allocated in a non-biased manner by dividing young, middle-aged, and elderly people into age groups, and participants who preferred the online option were given a uniform resource locator for the survey file; those who preferred an offline survey were provided with the structured questionnaire The survey utilized a self-completion method, and signed consent forms were obtained online and offline prior to the start of the survey. To prevent multiple submissions, the online survey environment was configured to allow only one response per participant after logging in with a Google account. Of the 244 completed surveys, the final 242 were used in the analysis after excluding two surveys (offline survey conducted among older adults) with insufficient responses.

### 5. Data analysis

The collected data were analyzed using the SPSS version 27.0 (IBM Corp., Armonk, NY, USA). To determine differences in demographics, health, and COVID-19 related characteristics according to age groups of study participants, we analyzed them using the Fisher-Freeman-Halton exact test. To determine the differences in COVID-19 related digital health literacy, COVID-19 anxiety syndrome, and hypochondriasis by age group, one-way analysis of variance was used, followed by post hoc analysis with Scheffe test. Multiple linear regression (enter method) was used to determine the effects of COVID-19 related digital health literacy and COVID-19 anxiety syndrome on hypochondriasis by age groups. The significance level for all statistical analyses was *p* < .05.

### 6. Ethical considerations

This study was reviewed and approved by the Institutional Review Board of the Cheongju University (IRB No.1041107-202206-HR-013-01). Participants were provided with an explanation of the purpose and methods of the study before beginning the survey. Those who voluntarily agreed to take part in the study were surveyed online and offline, using the method of their choice. Participants were informed in advance that the survey would be anonymous and confidential, that they could voluntarily stop participating at any time during the survey, and that they would not be penalized in any way. No personally identifiable information was collected during data collection, and research-related data was kept anonymized.

## RESULTS

### 1. Differences in socio-demographic characteristics according to age groups

The final participants consisted of 97 young adults (online: 95, offline: 2), 72 middle-aged adults (online: 41, offline: 31), and 73 older adults (online: 5, offline: 68). The young adult participants were 19~44 years old with a mean age of 25.89 ± 7.30, middle-aged participants were 45~64 years old with a mean age of 53.26 ± 5.82, and older adult participants were over 65 years old with a mean age of 72.26 ± 5.25. Analyses of the demographic characteristics according to age groups in this study revealed significant differences in sex, education level, marital status, occupation, and living arrangement (Table 1).

### 2. Differences of health and COVID-19 related characteristics according to age groups

As a result of analyzing the differences in health and COVID-19 related characteristics according to age groups, we found significant differences in chronic disease diagnosis, COVID-19 testing experience, and COVID-19 self-isolation experience (Table 2).

### 3. Differences of COVID-19 related digital health literacy, COVID-19 anxiety syndrome, and hypochondriasis according to age groups

There were significant differences in COVID-19 related digital health literacy (F = 31.30, *p* < .001) and hypochondriasis (F = 26.95, *p* <. 001) according to age groups. COVID-19 related digital health literacy levels were highest among young adults and lowest among older adults. There were significant differences between age groups in the following subscales of digital health literacy: searching (F = 37.52, *p* < .001), expressing (F = 19.85, *p* < .001), evaluating (F = 8.88, *p* < .001), and using (F = 31.05, *p* <. 001). Younger and middle-aged people scored similarly on the “evaluating” and “using” components of digital health literacy, but were higher than the older people.

The level of hypochondriasis was higher among middle-aged people compared to the younger and older people. Hypochondriasis differed significantly by age groups in most subscales: worry about illness (F = 16.05, *p* < .001), hypochondriacal beliefs (F = 9.51, *p* < .001), thanatophobia (F = 9.07, *p* < .001), disease phobia (F = 14.67, *p* < .001), bodily preoccupations (F = 15.95, *p* < .001), treatment experiences (F =23.06, *p* < .001) and effects of symptoms (F = 11.36, *p* < .001); middle-aged people had higher hypochondriasis scores than younger and older people. Young adults scored lower than middle-aged adults on health habits (F = 3.98, *p* = .020). They also scored lower than middle-aged and older adults on the treatment experiences (F = 23.06, *p* < .001), while middle-aged adults scored higher than older adults on the treatment experiences. However, there were no significant differences between age groups regarding COVID-19 anxiety syndrome (Table 3).

### 4. Effect of COVID-19 related digital health literacy and COVID-19 anxiety syndrome on hypochondriasis according to age groups

Among young adults, only COVID-19 anxiety syndrome (β = 0.28, *p* = .009) was found to have a significant effect on hypochondriasis, and this model was found to have no statistically significant explanatory power. Among middle-aged adults, COVID-19 anxiety syndrome (β = 0.47, *p* < .001) and digital health literacy related to information searching (β = −0.42, *p* =.018) were found to have a statistically significant effect on hypochondriasis. The explanatory power of this model was approximately 29% (F = 3.12, *p* = .001). Among older adults, COVID-19 anxiety syndrome (β = 0.40, *p* < .001) and having a diagnosis of a chronic disease (β = 0.32, *p* = .006) were identified as significant factors influencing hypochondriasis. The explanatory power of this model was 24% (F = 2.58, *p* = .006). COVID-19 anxiety syndrome was identified as a significant predictor of hypochondriasis across all age groups, with differences in the factors influencing hypochondriasis across age groups (Table 4).

## DISCUSSION

The study found that middle-aged and older adults had lower levels of COVID-19 related digital health literacy than young adults. These findings are consistent with previous studies that have found differences in digital health literacy levels according to age groups [30,31]. We thus found a generational gap in COVID-19 related digital health literacy. This gap varies depending on the point in the lifecycle at which digital technologies penetrate society [32]. Young adults, Millennials and Generation X, were exposed to technology as it was being created, and became central to their lives, while middle-aged Baby Boomers, now older adults, and the Silent Generation, were exposed to new technologies after their social and cultural identities were established, making them late adopters of innovation [32]. Digital health literacy influences the utilization of online-based health services [33,34], and the use of digital health information is also closely linked to changes in health behaviors and health statuses [35,36]; therefore, improving digital health literacy levels may influence future health outcomes.

In this study, health anxiety levels were higher among middle-aged adults at 48.8, compared to 33.0 among young adults and 32.9 among older adults. These findings are similar to previous studies that reported higher levels of hypochondriasis among those aged 30 years and older compared to those under 30 years [37], and higher levels of health anxiety among those aged 30-59 years compared to those under 30 years and those aged 60 years and older [38] due to COVID-19. In the current Korean society, middle-aged people are faced with the dual burden of supporting their parents and raising children [39], and they are worried and anxious about the emotional and financial distress of their children and other family members in the event of their untimely death [40]. Middle-aged adults experience more anxiety and fear of death than younger adults [41]. This explains the high level of health anxiety among middle-aged Koreans in the post-COVID-19 era. Thus, our findings suggest that health anxiety among middle-aged adults in the post-COVID-19 era deserves focused attention.

In this study, COVID-19 anxiety syndrome was a significant predictor of hypochondriasis in both middle-aged and older adults. Previous studies have identified positive correlations between health anxiety and online information seeking [42], and between COVID-19 anxiety and cyberchondria [43]. These results suggest that higher levels of COVID-19 related anxiety may lead to more excessive and persistent use of the internet to search for health information. Excessive information acquisition via the internet can lead to a vicious cycle of hypochondriasis, especially when health concerns are expressed in the form of cyberchondria in young adults who are highly dependent on the internet [22]. Therefore, it is necessary for governments and medical professionals to provide accurate medical information through online platforms such as social media, which are mainly accessed by young people, and to monitor inappropriate information online.

Digital health literacy, particularly in the domain of information searching, has been identified as a significant determinant of hypochondriasis among middle-aged individuals, in addition to the impact of COVID-19 anxiety syndrome. Previous studies have shown that online health information-seeking behavior is related to health literacy in middle-aged adults [44], that e-health information-seeking promotes positive health perceptions [45], and that online health information-seeking influences health anxiety [42]. Considering that middle-aged adults experience the highest levels of hypochondriasis, interventions designed to enhance online health information-seeking skills for middle-aged adults in the digital era could serve as a valuable approach to alleviating health anxiety.

In the older people, chronic diseases was also identified as significant predictor of hypochondriasis. Our finding, that chronic illness had a significant effect on hypochondriasis, is consistent with previous studies showing that chronic illness is associated with COVID-19 related health anxiety and hypochondriasis [38]. COVID-19 patients with chronic conditions are at an increased risk for serious and fatal health outcomes, age-related comorbidities are likely to increase death anxiety among older adults, especially those with chronic conditions, during the pandemic. Death anxiety is highly prevalent among older adults and is strongly associated with poor psychological well-being and challenges to successful aging, particularly among older adults with chronic conditions [46]. Older adults who have greater difficulty tolerating uncertainty may be more vulnerable to anxiety, and fear of death and illness may increase as they have more opportunities to encounter death, especially in later life [47]. Therefore, addressing hypochondriasis in older adults with chronic disease may require interventions that help individuals confront death-related uncertainty and build greater tolerance for such uncertainty.

Hypochondriasis not only increases the economic costs of increased healthcare utilization, but also affects mortality [48,49], and therefore requires attention from health professionals. The economic burden of hypochondriasis has been shown to range from 857.19 to 21137.55 USD per capita per year [48], and compared to people without hypochondriasis, those who have the condition are more likely to die from all causes [49]. These results demonstrate the paradox that despite the fear of illness and death, hypochondriasis actually increases the risk of mortality in people with hypochondriasis, and suggest that if COVID-19-induced hypochondriasis persists long after the pandemic is over, it may have implications for future mortality, in addition to increasing the economic burden.

This study aimed to build on lessons learned from the COVID-19 pandemic to lay the foundation for developing strategies to effectively respond to future emerging infectious disease outbreaks. This study is significant in that it identifies age-specific factors that influence health anxiety due to COVID-19, providing a foundation for developing age-specific and customized psychological health intervention strategies. The findings emphasize the importance of developing age-specific anxiety syndrome management strategies and access to digital health information to effectively manage health anxiety during future infectious disease epidemics. First, systems should be developed to monitor psychological conditions such as anxiety associated with emerging infectious diseases and to support all age groups during such crises. Second, educational programs should be implemented to improve digital health literacy, with a focus on skills to find accurate information online through the use of digital media, especially among digital migrants. Thirdly, special attention should be paid to helping vulnerable health groups address their health concerns through informed health outreach.

However, this study also has some limitations. First, the study was conducted under the special circumstances of the COVID-19 pandemic and recruited participants by age group; hence, this study used convenience sampling, a non-probability sampling method, to collect data, which may limit the generalizability of the results as the sample may not be representative of the entire study population. To address for this limitation, future studies should consider sampling methods to increase the representativeness of the sample. Second, the cross-sectional design of the study makes it hard to decide causal connections between study variables, and it is challenging to decide the long-term effects of COVID-19 anxiety syndrome and hypochondriasis triggered by COVID-19. Therefore, we suggest a longitudinal study of COVID-19 anxiety syndrome and hypochondriasis after the COVID-19 pandemic to determine the psychosocial impact of the extended coronavirus. Thirdly, this study used a mixed-methods data collection design, combining digital and non-digital methods to account for the COVID-19 situation and the preferences of individuals and age groups, but potential mode effects cannot be ruled out. However, we tried to maintain the same question format and content in each mode to reduce bias and ensure data consistency across modes. In addition, this study used translated instruments to collect data, which could potentially affect validity. To compensate for this, we attempted to ensure linguistic and cultural validity through back-translation and expert review. However, despite these efforts, we cannot rule out loss of meaning or differences in cultural context that may occur during the translation process. Future research should more comprehensively address cultural differences in the use of translated instruments to further validate their validity.

## CONCLUSIONS

This is a novel study that identifies the influencing factors of COVID-19-provoked hypochondriasis in terms of COVID-19 related digital health literacy and COVID-19 anxiety according to age groups in Korea. This study confirms that COVID-19 anxiety syndrome affects health concerns across all age groups, and suggests that COVID-19 response strategies should focus not only on the psychological problems caused by COVID-19, but also on strategies for coping with COVID-19. Based on the results of this study, it is necessary to establish age-differentiated health management strategies for health anxiety, including strategies to manage anxiety and improve digital health literacy in the context of emerging infectious diseases. Especially, in middle-aged adults, digital health literacy related to information searching was identified as a contributing factor to hypochondriasis; Highlighting the need for educational interventions to improve their ability to seek accurate health information online. In older adults, health conditions such as chronic diseases were found to affect hypochondriasis among older adults, suggesting that hypochondriasis in vulnerable age groups needs to be managed intensively.

## Figures and Tables

**Table 1.** Differences in Socio-demographic Characteristics According to Age Groups (N = 242)

Characteristics				*p* ^†^
	Young adults (19~44 years)	Middle-aged adults (45~64 years)	Older adults (≥ 65 years)	
	(n = 97)	(n = 72)	(n = 73)	
Online/offline survey	95 (97.9)/2 (2.1)	41 (56.9)/31 (43.1)	5 (6.8)/68 (93.2)	
	97 (40.1)	72 (29.7)	73 (30.2)	
Age (year)	25.89 ± 7.30	53.26 ± 5.82	72.26 ± 5.25	
Sex				
Men	21 (21.6)	36 (50.0)	34 (46.6)	< .001
Women	76 (78.4)	36 (50.0)	39 (53.4)	
Education level				
≤ Junior high school	0 (0.0)	5 (6.9)	34 (46.6)	< .001
High school	56 (57.7)	27 (37.5)	17 (23.3)	
≥ Bachelor’s degree	41 (42.3)	40 (55.6)	22 (30.1)	
Marital status				
Married	18 (18.6)	59 (81.9)	63 (86.3)	< .001
Unmarried & single (divorced, bereaved, separated, etc.)	79 (81.4)	13 (18.1)	10 (13.7)	
Employment				
Employed	39 (40.2)	63 (87.5)	26 (35.6)	< .001
Unemployed	58 (59.8)	9 (12.5)	47 (64.4)	
Living arrangement				
Single-person household	22 (22.7)	9 (12.5)	34 (46.6)	< .001
Multi-family household	75 (77.3)	63 (87.5)	39 (53.4)	
Economic status				
High (≥ middle-high)	29 (29.9)	13 (18.1)	21 (28.8)	.414
Middle	47 (48.5)	43 (59.7)	35 (47.9)	
Low (≤ middle-low)	21 (21.6)	16 (22.2)	17 (23.3)	

Values are presented as the mean ± standard deviation or n (%). ^†^Fisher-Freeman-Halton exact test.

**Table 2.** Differences in Health and COVID-19 Related Characteristics According to Age Groups (N = 242)

Characteristics				*p* ^†^
	Young adults (19-44 years)	Middle-aged adults (45-64 years)	Older adults (≥ 65 years)	
	(n = 97)	(n = 72)	(n = 73)	
Diagnosis of chronic disease				
Yes	17 (17.5)	23 (31.9)	31 (42.5)	.001
No	80 (82.5)	49 (68.1)	42 (57.5)	
Subjective health status				
Very good	11 (11.3)	6 (8.3)	6 (8.2)	.905
Good	26 (26.8)	18 (25.0)	15 (20.5)	
Fair	48 (49.5)	39 (54.2)	44 (60.3)	
Poor/Very poor	12 (12.4)	9 (12.5)	8 (11.0)	
COVID-19 testing experience				
Yes	88 (90.7)	56 (77.8)	43 (58.9)	< .001
No	9 (9.3)	16 (22.2)	30 (41.1)	
History of confirmed COVID-19 infection				
Yes	52 (53.6)	35 (48.6)	27 (37.0)	.095
No	45 (46.4)	37 (51.4)	46 (63.0)	
Experience of the after-effects of COVID-19				
Yes	17 (17.5)	13 (18.0)	12 (16.5)	.220
No	35 (36.1)	22 (30.6)	15 (20.5)	
Not applicable	45 (46.4)	37 (51.4)	46 (63.0)	
COVID-19 self-isolation experience				
Yes	66 (68.0)	37 (51.4)	31 (42.5)	.003
No	31 (32.0)	35 (48.6)	42 (57.5)	
Family members who died of COVID-19				
Yes	1 (1.0)	1 (1.4)	5 (6.8)	.086
No	96 (99.0)	72 (98.6)	68 (93.2)	

Values are presented as n (%). COVID-19 = Coronavirus disease 2019. ^†^Fisher-Freeman-Halton exact test.

**Table 3.** Differences in COVID-19 Related Digital Health Literacy, COVID-19 Anxiety Syndrome and Hypochondriasis According to Age Groups (N = 242)

Characteristics				Scheffe test	F (*p*)
	Young adults^a^	Middle-aged adults^b^	Older adults^c^		
		
Digital health literacy^†^	2.86 ± 0.48	2.64 ± 0.57	2.21 ± 0.57	a > b, a > c,	31.30 (< .001)
				b > c	
Searching	3.02 ± 0.55	2.74 ± 0.68	2.16 ± 0.71	a > b, a > c,	37.52 (< .001)
				b > c	
Expressing	2.91 ± 0.66	2.65 ± 0.63	2.27 ± 0.69	a > b, a > c,	19.85 (< .001)
				b > c	
Evaluating	2.59 ± 0.61	2.49 ± 0.62	2.20 ± 0.61	a > c, b > c	8.88 (< .001)
Using	2.92 ± 0.53	2.70 ± 0.61	2.20 ± 0.66	a > c, b > c	31.05 (< .001)
COVID-19 anxiety syndrome	13.53 ± 7.28	16.00 ± 8.60	13.56 ± 7.89		2.47 (.087)
Anxiety: perseveration	10.35 ± 5.17	12.00 ± 5.94	10.12 ± 5.54		2.57 (.079)
Anxiety: avoidance	3.18 ± 2.77	4.00 ± 3.27	3.44 ± 3.37		1.48 (.231)
Hypochondriasis^†^	33.04 ± 13.75	48.81 ± 19.35	32.85 ± 12.68	a < b, b > c	26.95 (< .001)
Worry about illness	6.70 ± 3.48	9.06 ± 4.18	5.63 ± 3.61	a < b, b > c	16.05 (< .001)
Health habits	4.72 ± 2.23	5.81 ± 2.73	4.84 ± 2.99	a < b	3.98 (.020)
Hypochondriacal beliefs	1.19 ± 1.80	2.44 ± 2.02	1.51 ± 1.87	a < b, b > c	9.51 (<. 001)
Thanatophobia	2.52 ± 2.80	4.01 ± 2.84	2.34 ± 2.17	a < b, b > c	9.07 (< .001)
Disease phobia	2.25 ± 2.70	4.51 ± 3.34	2.37 ± 2.72	a < b, b > c	14.67 (< .001)
Bodily preoccupations	4.49 ± 2.91	7.19 ± 3.73	4.86 ± 3.09	a < b, b > c	15.95 (< .001)
Treatment experiences	8.14 ± 3.05	12.10 ± 4.14	9.84 ± 4.14	a < b, a < c,	23.06 (< .001)
				b > c	
Effects of symptoms	3.03 ± 3.30	3.68 ± 2.88	1.47 ± 2.26	a < b, b > c	11.36 (< .001)

Values are presented as the mean ± standard deviation. COVID-19 = Coronavirus disease 2019.

† Scheffe test.

**Table 4.** The Effect of COVID-19 Related Digital Health Literacy and COVID-19 Anxiety Syndrome on Hypochondriasis According to Age Groups (N = 242)

Variables	Young adults				
	Hypochondriasis				
	B	SE	*β*	t	*p*
(Constant)	38.66	11.14		3.47	<.001
Digital health literacy-searching	−2.49	3.97	−0.10	−0.63	.532
Digital health literacy-expressing	0.37	3.22	0.02	0.11	.909
Digital health literacy-evaluating	0.96	3.17	0.04	0.30	.763
Digital health literacy-using	−3.45	4.28	−0.13	−0.81	.422
COVID-19 anxiety syndrome	0.53	0.20	0.28	2.66	.009
^†^Sex(1 = Women)	3.24	3.70	0.10	0.88	.383
^†^Education level (1 = high school)	−0.48	6.05	−0.02	−0.08	.937
^†^Marital status (1 = unmarried & single)	−7.05	4.36	−0.20	−1.62	.110
^†^Employment (1 = unemployed)	3.43	6.15	0.12	0.56	.578
^†^Living arrangement (1 = single-person household)	2.01	3.66	0.06	0.55	.584
^†^Diagnosis of chronic disease (1 = yes)	2.46	3.77	0.07	0.65	.515
^†^COVID-19 testing experience (1 = yes)	2.00	5.30	0.04	0.38	.707
^†^COVID-19 self-isolation experience (1 = yes)	−0.05	3.39	−0.01	−0.02	.988
*Adj R^2^*	0.04				
F(*p*)	1.29 (.234)				
**Variables**	**Middle-aged adults **				
	**Hypochondriasis**				
	**B**	**SE**	***β***	**t**	***p***
(Constant)	33.19	13.12		2.53	.014
Digital health literacy-searching	−12.01	4.91	−0.42	−2.45	.018
Digital health literacy-expressing	5.84	6.44	0.19	0.91	.369
Digital health literacy-evaluating	−5.71	5.95	−0.18	−0.96	.341
Digital health literacy-using	8.71	7.23	0.27	1.21	.233
COVID-19 anxiety syndrome	1.05	0.26	0.47	4.01	<.001
^†^Sex (1 = Women)	1.30	4.88	0.03	0.27	.791
^†^Education level (1 = ≤ junior high school)	3.57	10.06	0.05	0.35	.724
^†^Education level (1 = high school)	−3.87	4.66	−0.10	−0.83	.410
^†^Marital status (1 = unmarried & single)	3.85	5.96	0.08	0.65	.521
^†^Employment (1 = unemployed)	5.42	6.60	0.09	0.82	.415
^†^Living arrangement (1 = single-person household)	3.62	7.55	0.06	0.48	.634
^†^Diagnosis of chronic disease (1 = yes)	8.54	4.56	0.21	1.87	.066
^†^COVID-19 testing experience (1 = yes)	3.05	5.67	0.07	0.54	.593
^†^COVID-19 self-isolation experience (1 = yes)	1.13	4.86	0.03	0.23	.817
*Adj R^2^*	0.29				
F(*p*)	3.12 (.001)				
**Variables**	**Older adults**				
	**Hypochondriasis**				
	**B**	**SE**	***β***	**t**	***p***
(Constant)	32.72	8.12		4.03	< .001
Digital health literacy-searching	−1.79	3.15	−0.10	−0.57	.573
Digital health literacy-expressing	−0.23	3.23	−0.01	−0.07	.945
Digital health literacy-evaluating	−3.12	3.44	−0.15	−0.90	.371
Digital health literacy-using	−0.47	3.70	−0.03	−0.13	.899
COVID-19 anxiety syndrome	0.64	0.18	0.40	3.64	<.001
^†^Sex (1 = Women)	0.35	2.86	0.01	0.12	.903
^†^Education level (1 = ≤ junior high school)	−5.01	4.16	−0.20	−1.21	.233
^†^Education level (1 = high school)	−5.30	4.13	−0.18	−1.29	.204
^†^Marital status (1 = unmarried & single)	−3.86	4.29	−0.11	−0.90	.372
^†^Employment (1 = unemployed)	4.76	3.07	0.18	1.55	126
^†^Living arrangement (1 = single-person household)	1.76	2.99	0.07	0.59	.558
^†^Diagnosis of chronic disease (1 = yes)	8.14	2.84	0.32	2.87	.006
^†^COVID-19 testing experience (1 = yes)	4.02	3.85	0.16	1.04	.302
^†^COVID-19 self-isolation experience (1 = yes)	−5.03	3.71	−0.20	−1.36	.181
*Adj R^2^*	0.24				
F(*p*)	2.58 (.006)				

COVID-19 = Coronavirus disease 2019; SE = Standard error.

† Dummy variable= sex (reference group = men); education level (reference group = ≥ bachelor’s degree); marital status (reference group = married); employment (reference group = employed); living arrangement (reference group = multi-family household); diagnosis of chronic disease (reference group = no); COVID-19 testing experience (reference group = no); COVID-19 self-isolation experience (reference group = no).

## Data Availability

The data that support the findings of this study are available from the corresponding author upon reasonable request.
